# Retraction: Conservation tillage improves productivity of sunflower (*Helianthus annuus* L.) under reduced irrigation on sandy loam soil

**DOI:** 10.1371/journal.pone.0272480

**Published:** 2022-08-17

**Authors:** The PLOS ONE Editors

The *PLOS ONE* Editors retract this article [1] because it was identified as one of a series of submissions for which we have concerns about authorship, competing interests, and peer review. We regret that the issues were not addressed prior to the article’s publication.

ASher, SUA, ASattar, MI, AM, AQ, and ATKZ did not agree with the retraction. MYA, ON, and KG either did not respond directly or could not be reached.
